# Modified wavelet analysis of ECoG-pattern as promising tool for detection of the blood–brain barrier leakage

**DOI:** 10.1038/s41598-021-97427-9

**Published:** 2021-09-16

**Authors:** Anastasiya Runnova, Maksim Zhuravlev, Rodion Ukolov, Inna Blokhina, Alexander Dubrovski, Nikita Lezhnev, Evgeniya Sitnikova, Elena Saranceva, Anton Kiselev, Anatoly Karavaev, Anton Selskii, Oxana Semyachkina-Glushkovskaya, Thomas Penzel, Jurgen Kurths

**Affiliations:** 1 Saratov State Medical University, B. Kazachaya str. 112, Saratov, 410012 Russia; 2 Saratov State University, Astrakhanskaya str. 83, Saratov, 410012 Russia; 3 Institute of Higher Nervous Activity and Neurophysiology of RAS, (IHNA&NPh RAS), Butlerova str. 5a, Moscow, 117485 Russia; 4 Saratov Branch of the Institute of RadioEngineering and Electronics of Russian Academy of Sciences, Zelyonaya str. 38, Saratov, 410019 Russia; 5 National Medical Research Center for Therapy and Preventive Medicine, Petroverigsky per. 10, Moscow, 101953 Russia; 6 Charite Universitatsmedizin Berlin, Chariteplatz 1, Berlin, 10117 Germany; 7 Potsdam Institute for Climate Impact Research, Telegrafenberg A31, Potsdam, 14473 Germany

**Keywords:** Computational biophysics, Blood-brain barrier, Computational neuroscience

## Abstract

A new approach for detection oscillatory patterns and estimation of their dynamics based by a modified CWT skeleton method is presented. The method opens up additional perspectives for the analysis of subtle changes in the oscillatory activity of complex nonstationary signals. The method was applied to analyze unique experimental signals obtained in usual conditions and after the non-invasive increase in the blood–brain barrier (BBB) permeability in 10 male Wistar rats. The results of the wavelet-analysis of electrocorticography (ECoG) recorded in a normal physiological state and after an increase in the BBB permeability of animals demonstrate significant changes between these states during wakefulness of animals and an essential smoothing of these differences during sleep. Sleep is closely related to the processes of observed changes in the BBB permeability.

## Introduction

Sleep is essential for keeping healthy brain functions and processes during sleep that are capable of protecting cerebral small vessels from injuries associated with leakage of the blood–brain barrier (BBB)^1–5^. The latest findings discovered the importance of deep sleep in regulation of brain fluid homeostasis and clearance of toxins from the brain^6–8^. The deficit of sleep contributes to accumulation of non-necessary metabolites and toxins, such as beta-amyloid in the brain, that may cause disruption of the BBB^6,9–11^. Currently, information about sleep helps in diagnostics and therapy of a large family of cerebral small vessel diseases (CSVD), including Alzheimer’s disease, Parkinson diseases, dementia, sclerosis^1,2,12^. However, it is not clear whether sleep derived parameters have a diagnostic value in CSVD. The methodology for extracting disease-related correlates of sleep processes has not been developed yet.

Analysis of sleep is based upon quantification and mathematical processing of recorded electrical brain activity. Electroencephalography (EEG) is the preferred method for recording electrical brain activity from the scalp surface (in humans) and from the brain surface is known as electrocorticography, ECoG (clinical studies in humans or in laboratory animals). The EEG method is convenient for basic and clinical research, because it is easy to use and a moderate cost method providing high spatial and temporal resolution of signals. EEG signals are of high quality, therefore, they can be directly underwent by various kinds of mathematical analysis, including nonlinear dynamics, physics and statistical processing; EEG signals could also be processed automatically within the framework of software systems. In the present report we demonstrate the applicability of an automated EEG processing system that detected subtle changes in brain activity correlated with physiological effects that occur after powerful sound stimulation.

A number of studies have indicated that the time-frequency structure of EEG/ECoG during sleep reflects changes in functioning of the BBB, and some time-frequency parameters of EEG/ECoG may be putative biomarkers of BBB leakage. Initially, Sharma and Dey showed a remarkable flattering of the EEG activity at the time of BBB opening, suggesting that neural activity can be affected by the BBB leakage^13^. Later, Kiviniemi et al proposed the direct-current EEG pattern as a marker of the mannitol-related BBB opening in patients with brain tumor^14^. In our preliminary work using wavelet analysis, we demonstrated a similarity of EEG dynamics and the BBB opening that may represent an informative marker of BBB disruption^15,16^. The link between sleep and changes in BBB permeability remain enigmatic for researchers. There is a hypothesis that sleep and the BBB opening are associated with similar activation of clearance of macromolecules and toxins from the brain^17,18^. Therefore, neuronal activity during sleep is expected to be similar to those during the BBB opening that we confirmed in our previous studies^15,16^. Thus, the effective diagnostics of BBB leakage based on the results of EEG analysis during sleep may be beneficial, for instance, it may open a new era for the development of breakthrough bed-side technologies of evaluation of BBB dysfunction as early biomarker of CSVD associated with BBB disruption.

The continuous wavelet transformation (CWT) is among efficient tools for a spectral analysis of neurophysiological data^19–23^. Here, in order to detect specific oscillatory patterns during sleep which are associated with changes in BBB permeability, we apply the CWT and a modified skeleton method in the ECoG recorded in freely moving healthy rats. With this method, we evaluate changes of electrical brain activity after the non-invasive BBB opening (OBBB) in adult male Wistar rats ($n = 10$). CWT analysis of ECoG signals, recorded on these rats in the normal state (ECoG1, ECoG3) and after auditory exposure (ECoG2), causing an increase in the BBB permeability^17,24,25^. Behavioral sleep (BS) was determined when an animal took a relaxed sleeping with closed or semi-closed eyes, and these periods were accompanied by ECoG synchronization in all channels. The electrical muscular activity (electromiography, EMG) was low. The waking state (AW) included passive and active wakefulness when an animal was in standing position, moved around the cage or was immobile. The period of AW is accompanied by theta-waves in occipital ECoG and desynchronization and high EMG. Our study is aimed toward determining specific ECoG frequency ranges in which statistically significant changes in brain activity occur when BBB was opened, and also to define specific oscillatory patterns in ECoG associated with opening of the BBB (the number and duration).

## Experimental data

### Methods

#### Animals

Experiments were performed in 10 male Wistar rats at the biological laboratory of Saratov State University (Saratov, Russia). The living conditions and experimental work in animals followed the recommendations in the ARRIVE guidelines 2.0^26^, were done in accordance with “the Guide for the Care and Use of Laboratory Animals” and local ethical guidelines, and it was approved by the Local Bioethics Commission of the Saratov State University.

Animals were kept in a light/dark environment with lights on from 8:00 to 20:00, temperature at 20 C, humidity 40-60% with standard food and tap water provided ad libitum. At the age of 5 months (body weight 250–280 g) rats were implanted with an optical window, polyethylene catheter and epidural electrodes for ECoG recording (see Appendix A.1).

#### Experimental design

ECoG was recorded three times. The first baseline recording session, ECoG1, lasted 1–1.5 h (between 2 and 6 pm). Next day after the ECoG1 recording, rats were exposed to audio stimulation consisting of 60 min loud sound (110 dB, 370 Hz) and during 2 h that is known to increase BBB permeability^24,25,27^ (see Appendix A.3). During this sound exposure, rats stayed in Plexiglas sound isolated chamber and the second ECoG was recorded immediately after the sound stimulation during 1 h (ECoG2). It is assumed^27,28^ that the abovementioned sound stimulation can lead to increased permeability of the BBB for 30–60 min. Two days after sound exposition, the third ECoG was recorded during 1–1.5 h (ECoG3). Recordings of ECoG1, ECoG2 and ECoG3 were done in the same way.

Two stages of the sleep-wake cycle were identified in ECoG: behavioral sleep and wakefulness (see Appendix A.2). So, the experimental data was divided into six types of records in each rat, namely, ECoG1-awake, ECoG1-sleep, ECoG2-awake, ECoG2-sleep, ECoG3-awake, ECoG3-sleep. A detailed analysis was carried out using the developed method to detect oscillatory patterns according to the each ECoG1-3 series.

**Figure 1:**
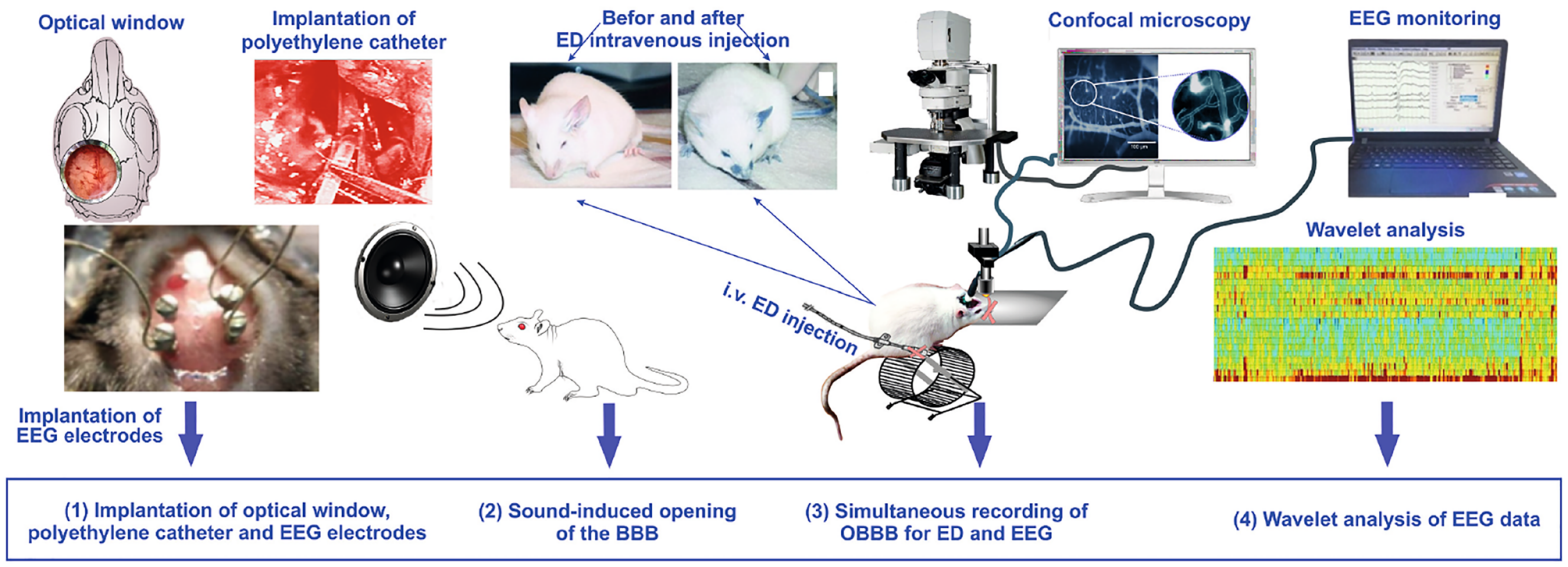
Schematic illustration of design of experiments: (1) optical window, polyethylene catheter and EcoG electrodes were implanted; (2) ten days afterward, registration of ECoG1; rats were underwent to the intermittent sound (100 dB, 370 Hz) during 2h (60 sec sound and 60 sec—pause); (3) 60 min sound-off, we performed in vivo real time fluorescent microscopy of the BBB opening for ED (i. v.), which we detected as bright fluorescence around the cerebral microvessels; (4) when the optical signs of ED leakage were detected, ECoG2 recordings were performed during 60 min; (5) the control rats decapitated and their brains were collected for confirmation of the BBB opening for ED using confocal microscopy; two day afterward, registration of ECoG3; (6) ECoG recordings were collected and analyzed using CWT approaches. The final collages were generated using the licensed program Colel Draw X8 (https://www.coreldraw.com).

Figure 1 illustrates main experimental steps: (1) implantation of stationar an optical window, polyethylene catheter and invasive ECoG electrodes; (2) registration of ECoG1, ECoG2, ECoG3 in rats ($n=10$); afterward, rats were underwent to the intermittent sound (100 dB, 370 Hz) during 2h (60 sec sound and 60 sec—pause) (see Appendix A.3); (3) 60 min sound-off, we performed in vivo real time fluorescent microscopy of the BBB opening for ED (i. v.), which we detected as bright fluorescence around the cerebral microvessels; (4) when we observed the optical signs of ED leakage, ECoG recordings in rats were performed during 60 min. The choice of time of ECoG recordings was related to our previous results discovering the window of sound-induced BBB opening that is 60–180 min after sound exposure^15,16,24,25^.

## Results

We use our model of loud sound-induced OBBB associated with an activation of the lymphatic clearance of macromolecules from the brain^15,16,24,25^. This model causes a reversible OBBB in 11 brain regions with a statistical significant increase in the BBB permeability to low and high weight molecular compounds for the short time period 1–4 h after music exposure without cochlear and brain injuries^24,25,27^.

**Figure 2:**
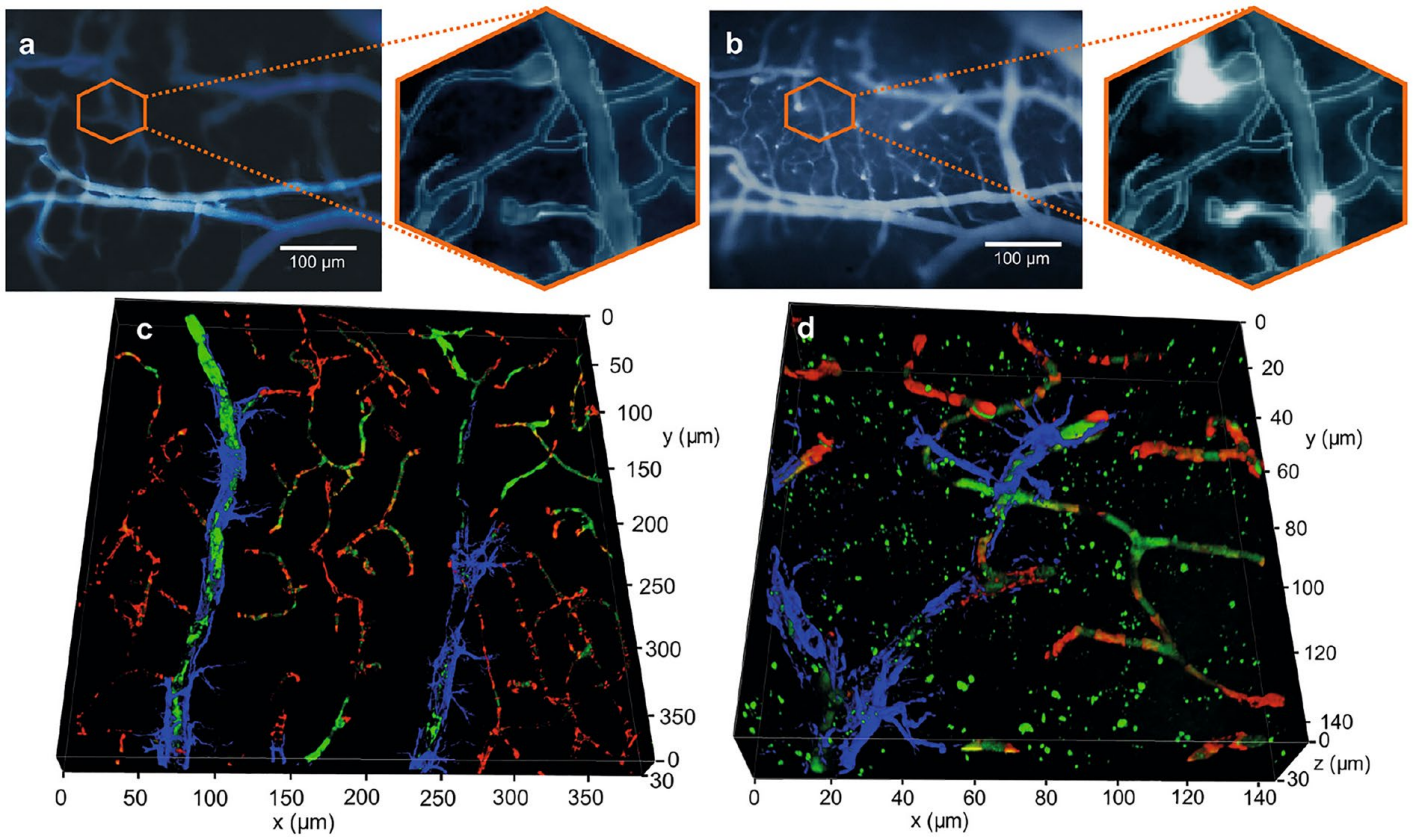
The sound-induced OBBB to the albumin complex of ED (68 kDa): (**a,b**)—in vivo real time fluorescent microscopy of the cerebral microvessels filled ED (no EB leakage) before music exposure (**a**) and EB extravasation from the cerebral capillaries into the brain tissues after music impact indicating OBBB (**b**); (**c**,**d**)—confocal imaging of brain slices in rats with the intact BBB (**c**) and with OBBB (**d**); the brain microvessels (red color) were labeled NG2, astrocytes (blue color) were labeled by GFAP; the extravasation of the albumin complex of ED (**b**,**d**) is shown as a bright fluorescence (**b**) around the cortex capillaries and green points (**d**) among the cerebral microvessels and astrocytes. In Fig., (**a**,**b**) panels were produced by microscope (Axio Imager A1, Zeiss, Germany) equipped with CMOS camera (acA1920-40uc, Basler AG, Germany), 10 $\times$ 0.3 objective lens and Evans Blue dye filter sets 49019 (Chroma, USA); (**c**,**d**) panels were imaged by confocal microscope (Leica SP5, Germany). The final collages were generated using the licensed program Colel Draw X8 (https://www.coreldraw.com).

To perform the EEG recordings during OBBB, we constructed an original design of experiments on the same rats. One hour after music exposure, OBBB was optically and non-invasively visualized using in vivo real time fluorescent microscopy of Evans Blue dye (ED) leakage via an optical window in awake behavior mice (Fig. 1). When we effectively detected OBBB as appearance of bright ED fluorescence around the cortical capillaries, the EEG were recorded in awake rats during 3h. Figure 2a,b illustrate typical images of the cerebral microvessels before and after OBBB to ED. Thus, ECoG and OBBB was simultaneous measured in the same rats. After ECoG recordings, to confirm OBBB, an additional ex vivo study of the BBB permeability to ED was performed using confocal microscopy. Figure 2c,d clearly demonstrate the confocal imaging of extravasation of ED from the cortex capillaries into the brain tissues that we did not observe in intact rats.

**Figure 3:**
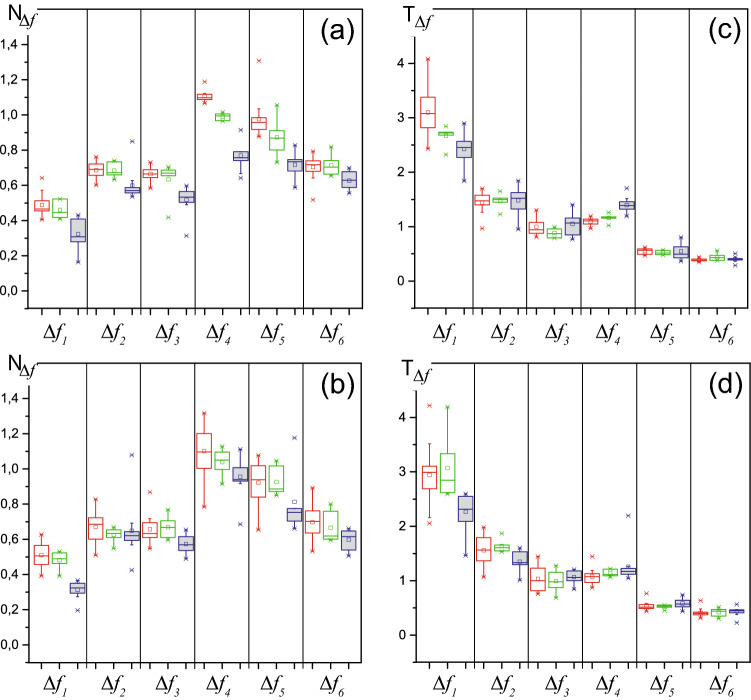
The diagrams present range of normalized number $N_{\Delta f}$ and duration $T_{\Delta f}$ of the oscillatory patterns for six selected frequency ranges for animal #3: (**a**,**b**) are the pattern’s normalized $N_{\Delta f}$ (??) for wakefulness and sleep periods, respectively, (**c, d**) are the pattern’s normalized durations $T_{\Delta f}$ (??) for similar animals states. For each frequency band $\Delta$(**f**), the results are obtained for the records ECoG1 (the first diagram in each band, highlighted in red), ECoG3 (the second diagram in each band, highlighted in green), ECoG2 (the third diagram in each band, highlighted in blue) . The horizontal line shows the median value, the square—the arithmetic mean, and the crosses “x”—single outliers on each diagram.

We use the method of evaluating patterns to analyze the features of oscillatory activity in ECoGs 1–3, presented in Appendix B. The number and duration of the oscillatory patterns for each ECoG recording are estimated (see Appendix C). The average frequency of each pattern is calculated. Each pattern is assigned by its average frequency to one of the six frequency intervals $\Delta f_1 [1; 2.5]$ Hz, $\Delta f_2 [2.5; 4.5]$ Hz, $\Delta f_3 [4.5; 6.5]$ Hz, $\Delta f_4 [6.5; 9]$ Hz, $\Delta f_5 [9; 12]$ Hz, $\Delta f_6 [12; 14]$ Hz. We estimate the normalized number and duration of the patterns in each frequency interval for each episode of sleep and wakefulness. Figure 3 presents the results of calculating the normalized number $N_{\Delta f}$ and duration $T_{\Delta f}$ of the oscillatory patterns for animal #3.

The results obtained from the wavelet-analysis of rat #3 are set out in Fig. 3. It is clearly seen that the number and duration of the oscillatory patterns are an extremely stable characteristic of brain activity. In the background ECoG recording before stimulation (ECoG1) during sleep and wakefulness, statistically significant differences in these parameters were not observed. This fact is due to the lack of amplitude ranking of the considered patterns. Thus, the oscillatory activity in the ECoG both during sleep and during wakefulness demonstrates a single “skeleton” of patterns, differing only in the fraction of energy falling on certain frequency ranges.

Immediately after the end of stimulation (ECoG2), the parameters of the oscillatory patterns during different behavioral states also do not differ statistically. However, a comparison of these parameters in the ECoG1 and ECoG2 records shows statistically significant differences for the number of patterns and less variation in their duration.

The parameters of the patterns in the ECoG3 recording exhibit that the activity of the brain returns to the background ECoG1 state two days after the sound stimulation practically in all frequency bands.

Further, we evaluate the statistical characteristics of the changes in oscillatory activity after auditory exposure in the all rats group.

Then, a statistical analysis is used to estimate changes in oscillatory activity after auditory exposure in the rats group. Changes in $N_{\Delta f}$ and $T_{\Delta f}$ in ECoG2 and ECoG1, ECoG3 are compared based on the characteristics $\delta _{N_{\Delta f}}$ and $\delta _{T_{\Delta f}}$ as follows:

1 $$\begin{aligned}&\delta _{N_{\Delta f} } =0.5\left( \frac{\left\langle N_{\Delta f} \right\rangle _{ECoG1} }{\left\langle N_{\Delta f} \right\rangle _{ECoG2} } +\frac{\left\langle N_{\Delta f} \right\rangle _{ECoG3} }{\left\langle N_{\Delta f} \right\rangle _{ECoG2} } \right) , \end{aligned}$$

2 $$\begin{aligned}&\delta _{T_{\Delta f} } =0.5\left( \frac{\left\langle T_{\Delta f} \right\rangle _{ECoG1} }{\left\langle T_{\Delta f} \right\rangle _{ECoG2} } +\frac{\left\langle T_{\Delta f} \right\rangle _{ECoG3} }{\left\langle T_{\Delta f} \right\rangle _{ECoG2} } \right) . \end{aligned}$$

**Figure 4:**
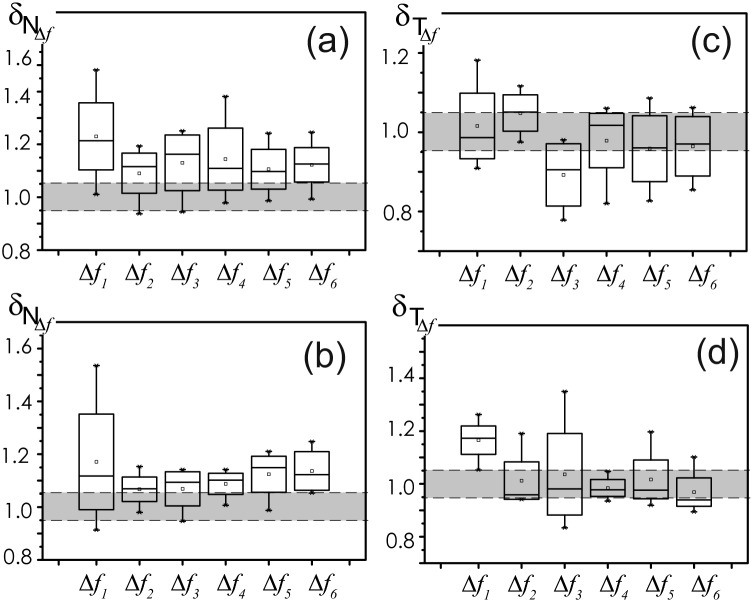
The results of a statistical assessment of the average number $N_{\Delta f}$ and the duration $T_{\Delta f}$ of oscillatory patterns *P* for a group of experimental animals: (**a**,**b**)—$\delta _{N_{\Delta f}}$ (1) for wakefulness and sleep states, respectively, (**c,d**)—$\delta _{T_{\Delta f}}$ (2) for the same animal conditions. Band of minor deviations $\delta$ [0.95; 1.05] is highlighted in grey.

The results of these evaluations in rats group are set out in Fig. 4. As can be seen there, we single out a zone of statistical estimates $\delta _{N_{\Delta f}}$ and $\delta _{T_{\Delta f}}$, close to unity, for which the characteristics on the records ECoG2 and ECoG1, 3 practically do not differ.

Figure 4 very clearly demonstrates the interesting complex situation of changes in the ECoG after a powerful auditory exposure. First of all, for awaking state, the comparing characteristic $\delta _{N_{\Delta f}}$ in ECoG2—awake almost all frequency bands is experiencing a growth, especially in region of “slow” activity in band $\Delta f_{1-3}$ (see Fig. 4a). At the same time, for the sleep state, in Fig. 4b there is a trend of analogous increasing $\delta _{N_{\Delta f}}$. Nonetheless, for this physiological state of rats, the region of “slow” activity in the bands $\Delta f_{1-3}$ shows less changes of the patterns number $N_{\Delta f}$ after auditory exposure than for wakefulness. Although the same statistical characteristic changes significantly in the frequency bands $\Delta f_{4-6}$. Note that we observe a decrease in the number of patterns $N_{\Delta f}$ compared to normal conditions of both sleep and wakefulness states during auditory stimulation (ECoG2).

At the same time, the duration $T_{\Delta f}$ of the oscillatory patterns behaves differently. In Fig. 4c, in the wakeful state, we observe a significant decrease in $\delta _{T_{\Delta f}}$ only for the frequency band $\Delta f_{3}$. In Fig. 4d, for the sleep state, the most peculiar is a increase in $\delta _{T_{\Delta f}}$ of the most “slow” patterns belonging to the frequency band $\Delta f_{1}$.

## Discussion

Thus, the presented method allows us to evaluate in detail changes in ECoG activity for rats in various conditions—normal, after auditory exposure and after the rehabilitation of animals after auditory exposure. The states of ECoG1 and ECoG3 do not change fundamentally, demonstrating only the usual natural variability for biological objects (Fig. 3). Interestingly, these changes for the wakefulness state, which is more associated with random fluctuations in external environmental signals, somewhat exceeded those for the sleep state.

However, an analysis of the state of ECoG2 after a powerful auditory stimulation and its comparison with normal states demonstrated several changes in the time-frequency structure of the electrical activity of the neural ensembles of the animal brain. We mainly find that:

(i) First of all, it is worth noting that the main changes are associated with varying the **number** of patterns of oscillatory activity on the ECoG. Only for the two frequency ranges $\Delta f_{1}$ ([1; 2.5] Hz) and $\Delta f_{3}$ ([4.5; 6.5] Hz) we can see a noticeable change in the time duration of these patterns. The auditory exposure leads to a rather mild change in the electrical activity of neural ensembles, correlated to or, possibly, directly caused by changes in BBB permeability and an increase in the current gradient in the lymphatic system of the animal’s brain.

(ii) Secondly, being in the natural physiological states of sleep and wakefulness, animals demonstrate significantly different mechanisms for changing brain activity on their ECoG during auditory exposure. A decrease in the number of patterns is observed in all considered frequency ranges of oscillatory activity and it is about 13 % during wakefulness after an auditory stimulation (i. e. in a state of increased BBB permeability). Moreover, the very structure of these patterns remains unchanged, except again for the oscillatory patterns in the frequency range $\Delta f_{3}$ ([4,5; 6,5] Hz). During sleep, the picture is less uniform. The oscillatory activity is least affected by the number of its patterns in the frequency ranges $\Delta f_{2}$ ([2.5; 4.5] Hz) and $\Delta f_{3}$ ([4.5; 6.5] Hz). Moreover, the decrease in the number of oscillatory patterns is about 8 %. For the time duration of the patterns, there is interest only for a decrease in the lifetime of the “slowest” oscillatory activity, the frequency of which does not exceed 2.5 Hz.

Recent work by Fultz^29^ has established that a connection between the “slow” oscillations of the brain electrical activity and physiological brain processes associated with variations in the blood–brain barrier permeability and brain drainage system. At the same time, we observe that an increase in BBB permeability caused by auditory exposure, can also cause changes in higher frequency activity for frequencies [4,5; 6.5] Hz. Also, the complex picture observed for the state of sleep is of undoubted interest.

We see smaller changes in the ECoG activity of the animal’s brain during sleep state in the normal state (ECoG1 and ECoG3) and after an auditory stimulation (ECoG2). In other words, during sleep, the response of the auditory effect on the animal’s brain observed on the ECoG is compensated by some internal processes. Possibly, the smaller amplitudes of changes after the auditory exposure in animals during sleep are an indirect evidence for the existence of so-called randomly occurring of increased activity of the brain lymphatic system in sleep stage natural time intervals. We can conclude that mild, non-invasive auditory stimulation weakly alters the recorded electrical activity of the brain during sleep.

The use of this approach for the analysis of ECoG in rats after an experimental increase in the BBB permeability enable us to study changes in the oscillatory ECoG activity of the brain. We have demonstrated that the BBB opening leads to weaker changes in the sleep state than in the waking state, which is a significant outcome. Obviously, such a results can be explained by the emergence of natural “windows” of increasing the BBB permeability during the state of sleep in animals. However, at the same time, we can assume that in the state of sleep we observe the activation of the mechanism for reducing the artificially induced BBB permeability.

## Conclusion

In this study, a new approach to numerically detect oscillatory patterns by means of the modified CWT skeleton method is presented. The proposed method opens up additional perspectives for the analysis of subtle changes in the ocillational activity of complex nonstationary signals. The article presents the approbation of this method to analyze unique experimental signals obtained in usual conditions and after the non-invasive increase in the BBB permeability in a group of rats.

We believe that further careful use of our proposed method for analyzing the ECoG activity of the brain may be useful for searching for weak effects of a natural increase in the activity of the lymphatic drainage system of the brain in a state of natural sleep. The detection of patterns characteristic of the state after auditory exposure during natural sleep of an animal (or a person) can become the basis for identifying for the characteristics of subtle physiological processes on the electrical activity of the brain.

## Supplementary Information

Supplementary Information.
