# Supplementary material for: Modified wavelet analysis of ECoG-pattern as promising tool for detection of the blood–brain barrier leakage
Source: Sci Rep. 2021 Sep 16;11:18505. doi: 10.1038/s41598-021-97427-9 (PMC8445940; doi:10.1038/s41598-021-97427-9)
Supplement: Supplementary file 1 — Supplementary Information. [file 41598_2021_97427_MOESM1_ESM.pdf]

Appendix A. Materials

Appendix A1. ECoG recording

Pinnacle Technology (Taiwan) was used to record two-channels ECoG/one channel electromyogram (EMG). During steretaxic operation, rats were kept under inhalation anesthesia (2% isoflurane at 1L/min N_2O/O_2 - 70:30) and were implanted with two silver electrodes (tip diameter 2-3 μm) at a depth of 150 μm inserted in right/left frontal cortex (L: 2.5 mm and D: 2 mm from Bregma). Small burr holes were drilled in the scull, ECoG wire leads were inserted in the epidural space and secured with dental acrylic. In order to control postoperative pain, Ibuprofen (15 mg/kg) was provided in drinking water during 2-3 days prior to surgery and more than 3 days after the surgery. Rats were allowed 10 days to recover from surgery prior to recording session.

Appendix A2. Sleep and wake states recognition

ECoG signals underwent a time-frequency transform. Wakefulness was defined as a desynchronized ECoG with low amplitude and high-frequency dynamics (> 10 %, $8-12$ Hz), NREM sleep as synchronized ECoG with high amplitude low-frequency delta activity ($0-4$ Hz) comprising > 30 % of ECoG waveforms/epoch, and REM by the presence of theta activity ($5-10$ Hz) comprising > 20 % of ECoG waveforms/epoch. Sleep/wakefulness states were detected using the method described in[?]. The NREM and REM stages were combined into behavioral sleep for further analysis. The typical sequence of sleep-wake states over a 1.5 h time interval is shown in Fig. 1.

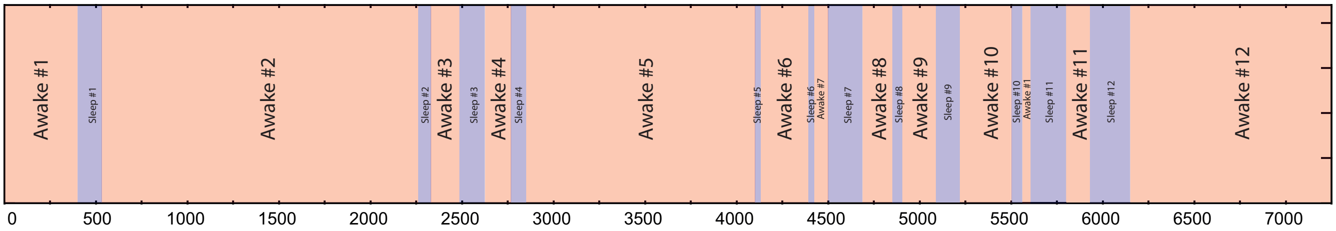

Figure 1. Sequence of sleep and wake states in rat # 2 in ECoG1. Periods of wakefulness are shown in pink ($n = 12$ episodes), and periods of sleep in blue ($n = 12$ episodes).

Appendix A3. Sound-induced opening of the BBB

Reversible and short-lasting opening of the BBB was induced using our original model of sound-related increased permeability of the BBB[?,?,?,?]. Sound stimulation (100 dB, 370 Hz) was provided via transducer (100 dB, 7A, 12 V, Auto VAZ PJSC, Tolyatti, Russia). The sound level was measured directly in a cage of animals using sound level meter (Megeon 92130, Russia). The duration of sound stimulation was 2 h in an altenating mode: 60 s – sound and 60 s – pause. In a series of preliminary experiments, we studied the dose and time effects of loud sound on the BBB opening to the Evans Blue Albumin Complex (EBAC, 68.5 kDa). The BBB opening by sound we performed in awake rat freely moving in the cage.

In vivo **real time fluorescent microscopy of extravasation of Evans Blue** from the cerebral vessels into the brain tissues was performed via optical window. Ten days before imaging, chronic optical windows (∅ 3 mm)[?] were prepared in coordinates 4 mm lateral to bregma and 3 mm medial on the acrylic platform for ECoG electrodes. In parallel, a polyethylene catheter (PE-10 tip, Scientific Commodities Inc., Lake Havasu City, Arizona) was inserted into the right femoral vein for ED intravenous injection in a single bolus dose (2 mg/100 g, 1% solution in physiological 0.9% saline). Microscope (Axio Imager A1, Zeiss, Germany) was equipped with CMOS camera (acA1920-40uc, Basler AG, Germany), 10×0.3 objective lens and Evans Blue dye filter sets 49019 (Chroma, USA). Continuous wave laser diode module (50000463, Laserlands.net, China) with 160 mW output power at 635 nm was used to excite the dye florescence. Laser beam was expanded with a cylindrical lens ($f = 50$ mm) and then directed towards an object at 45 $\circ$ with respect to the microscope optical axis. To reduce the sample irradiation, the laser was synchronized with the camera "fire" output to turn it on only for the image capturing period. The awake rats were positioned at the microscope stage using 3D printed homemade system and images were recorded for 5 minutes at a frame rate of 1 frame per second.

Confocal microscopy was performed to confirm an increased BBB permeability to ED after ECoG recordings. Rats were decapitated and the brains were quickly removed and fixed with 4% neutral buffered formalin for 24 h, cut into 50-μm thick slices on a vibratome (Leica VT 1000S Microsystem, Germany).The goat anti-mouse NG2 antibody (1:500; ab 50009, Abcam, Cambridge, United Kingdom) and goat anti-rabbit GFAP antibody (1:500; ab 207165, Abcam, Cambridge, United Kingdom). After several rinses in PBS, the slides were incubated for 1 hour with 130 μl fluorescent-labeled secondary antibodies (goat anti-mouse IgG (H+L) Alexa Four 647; goat anti-rabbit IgG (H+L) Alexa Four 488; Invitrogen, Molecular Probes, Eugene,

Oregon, USA). The slices were analyzed using a confocal microscope (Leica SP5, Germany). Approximately $8-12$ slices per animal from cortical and subcortical (excepting hypothalamus and choroid plexus where the BBB is leaky) regions were imaged.

Appendix B. Method of Data Analyse

We propose here a new method to detect oscillatory patterns in complex nonstationary signals. The mathematical basis of the developed method is the CWT[?, ?, ?]:

$$W(s,t) = \sqrt{s}\int_{-\infty}^{\infty} x(t)\psi^{*}(\frac{t-t_0}{s})dt \tag{1}$$

where $x(t)$ is the analyzed signal, and s is the time scale that determines the wavelet width, "*" is the complex conjugation, and $\psi_{t_0,s}(t)$ is the basis of the wavelet transform in the form of a complex function. In the framework of working with biological signals, a Morlet wavelet[?] is traditionally used as the basis function:

$$\psi_{t_0,s}(t) = \sqrt{f}\pi^{\frac{1}{4}} e^{j\omega_0 f(t-t_0)} e^{\frac{f(t-t_0)^2}{2}}, \tag{2}$$

where $\omega_0 = 2\pi$ is the wavelet scaling parameter that provides a relationship between the time scale of the wavelet transform (s) and the Fourier transform frequency (f), where $f = 1/s$. Thus, using the Morlet wavelet, we can work with the usual classical frequency representation of signals when calculating CWT.

The main advantages of wavelet analysis are the simplicity of the time-frequency sweep of noisy non-stationary signals and the good speed of numerical processing. CWT makes it possible to clearly and accurately trace the dynamics of the dominant components with a maximum frequency in the signal. However, if it is necessary to consider the dynamics at the accompanying frequency ranges. A problem arises in identifying various patterns of activity that exist at the same time on the original complex signals. In biological systems like neural ensembles, different patterns of activity often simultaneously develop[?, ?]. Even in model systems, as coupled neural ensembles, it was demonstrated the presence of several synchronous modes that switch when the nature of the connection changes[?]. Apparently, in the study of recorded brain activity, we can observe similar processes, which, however, are very difficult to detect. A simple assessment of the presence and amount of oscillatory activity in different frequency ranges substantially aggravates the situation and, possibly, complicates the task of assessing the dynamics of processes actually occurring in a biological system, and e.g., reducing the quality of the work of neurointerface devices.

In the framework of applying CWT, in particular, the skeleton CWT method[?, ?, ?] is used to improve the quality of assessment of such coexisting processes. This technique is based on the identification in the analyzed frequency range of the local maximum in the instantaneous distribution of CWT energy at every time. The following relation determines the instant CWT-energy distribution:

$$E(f,t_n) = |W(f,t_n)|^2. \tag{3}$$

Fig. 3, *a* illustrates the algorithm of the classical skeleton method, according to which a set of local extrema of function (3), can be identified for each time moment t_n. In other words, for each moment t_n there will be a set of frequencies f_i, $i = 1,2,\dots k$, where the first skeleton is observed at the frequency f_1, i. e., the maximum amplitude of the instantaneous energy CWT (3), $E(f_1,t_n) = \max[E(f,t_n)]$. For the following frequencies $f_2 f_3,\dots,f_k$, the value of relation (3) decreases, i. e., $E(f_1,t_n) > E(f_2,t_n) > E(f_3,t_n) > \dots > E(f_k,t_n)$. This technique significantly reduces the time of numerical calculations. It also simplifies further analysis in the time-frequency domains, because this approach does not give a large part of the secondary information. Usually the analysis of ECoG and/or EEG activity is limited to considering the first three CWT skeletons ($k = 3$)[?, ?, ?]. This technique is aimed totally at detecting the dominant frequency components for each specific time moment.

We propose a new method by an improved CWT skeleton approach to gain a detailed understanding of oscillatory patterns dynamics in complex non-stationary signals. The first feature of the developed method is the absence of ranking by the amplitude value of the instantaneous energy $E(f,t_n)$ for a set of frequencies f_i corresponding to the local maximum of $E(f_i,t_n)$. The second feature of the developed method is to take into account the entire set of the detected local maximum ("skeletons") in an entire frequency range. These features determine the advantages of this approache, and, in particular, make it possible to detect all the individual oscillatory patterns being at each moment in signal. Moreover, this method offers an effective way to estimate the dynamics of the oscillatory patterns with the automatic calculation of changes in the average frequency and amplitude of a pattern. The algorithm of the developed method to detect oscillatory patterns is described below. We can describe the algorithm of this approache in the following steps.

Step 1. In each time moment t_n, we compose a set of frequencies f_j, where $j = 1,2,\dots,m$, which correspond to the local maximum $E(f_j,t_n)$ of the instantaneous CWT-energy. The sequence number j characterizes only the sequence number of the

extrema and is not related to the amplitude $E(f_j, t_n)$. Thus, in the process of analyzing the total duration of the studied signal, a set of frequencies f_j^n is formed, where n is the duration of the experimental signal, i. e., the number of time samples in the signal.

Step 2. We denote the condition for the development of an activity pattern with a frequency f_j. To do this, we consider the following condition on each time interval $[t_n; t_{n+1}]$ for each frequency f_j:

$$\left|f_j^n - f_s^{n+1}\right| < \delta, \tag{4}$$

where f_j^n is a set of frequencies for which the local maximum $E(f_j, t_n)$ (3) observed at time step t_n, and f_j^{n+1} are similar set of frequencies with local maximum $E(f_j, t_{n+1})$ for the next time step t_{n+1}, δ is a numerical constant. The choice of the value of δ-constant is based on the signal sampling frequency and exceeds it by 1-2 orders of magnitude, which allows minimizing the loss of information about the frequency patterns existing in the signal under study and reducing the influence of the signal's numerical noise.

In step 3 step of the algorithm, we test a condition (4). If condition (4) is satisfied for some frequencies $f_{(a1)^n}$ and $f_{(a2)^{n+1}}$, then the activity at these frequencies in the time interval $[t_n; t_{n+1}]$ can be regarded as the development of one oscillatory pattern. We then denote the frequency data $f_{(a1)^n}$ and $f_{(a2)^{n+1}}$ as $(a1)$ and $(a2)$, respectively. Next, for frequency $(a2)$, we again analyze (4) for the next time step t_{n+2}. If the condition is satisfied for a given time step, then the identified pattern will continue further with a certain frequency $(a3)$.

The described actions must be repeated cyclically until the moment when condition (4) becomes incorrect, in other words, until the end of the activity of this oscillatory pattern. Thus, each oscillatory pattern P can be described by the frequency at each time moment of its existence, i. e., $P(f,t) = \{\{(a1), t_n\}, \{(a2), t_{n+1}\}, \ldots, \{(am), t_{n+m}\}\}$, where m characterizes the time duration of pattern "life". Then the time duration of the pattern P can be defined as

$$\tau = t_{n+m} - t_n, \tag{5}$$

and for the case of equidistant experimental time series, we use the expression $\tau = m\Delta t$, where Δt is the sampling time interval. So, the average frequency f_{md} can be estimated for each frequency pattern P as

$$f_{md} = \sum_{i=1}^{m} (ai)/m. \tag{6}$$

For further analysis, we denote the following selection criterion for the correct oscillatory patterns P. If the time duration τ of the pattern P does not exceed the oscillation period of its average frequency f_{md}, i. e., $\tau < (f_{md})^{-1}$, then this pattern must be considered a random noise interference and should not be taken into account in the further analysis of the signal.

To demonstrate the improved detection for time of various oscillatory patterns in the signal, we apply the proposed method to a concrete elementary model of experimental nonstationary signal. Figure 2, (a) shows a temporary realization of a model signal in the following form:

$$x(t) = (A_2(t) \cdot sin(2\pi\omega_2 t - \pi) - A_1(t) \cdot sin(2\pi\omega_1 t - \pi/2)) + A_3(t) \cdot sin(2\pi\omega_3 t), \tag{7}$$

where $\omega_1 = 39.5$ Hz for the time interval $0 - 17.9$ seconds, and already for the time interval $24.5 - 50.0$ seconds $\omega_1 = 38.5$ Hz, the frequency value for different time intervals also varied, for the time interval $0 - 7$ seconds $\omega_2 = 21.6$ Hz, for an interval of $15.5 - 29.0$ seconds $\omega_2 = 20.0$ Hz and for the interval $42.5 - 55$ seconds $\omega_2 = 22.5$ Hz, the frequency of the third sine remained unchanged over the entire time interval and had the following value $\omega_3 = 9.35$ Hz. Parameters $A_1(t)$, $A_2(t)$ and $A_3(t)$ are the amplitudes of the sines in the model system. At each moment of time the amplitude values determine the intensity of the various oscillatory components (patterns) and, ultimately, the form and amplitude of the entire signal $x(t)$. The amplitudes $A_1(t)$, $A_2(t)$ and $A_3(t)$ are highlighted with colors in Fig. 2, (b). The time dependences of the amplitudes $A_1(t)$ and $A_2(t)$ show the time intervals of zero values (highlighted by gray rectangles in Fig. 2, (b)), at which the corresponding oscillatory components are not present in the signal $x(t)$.

The first CWT - skeleton allows to detect only the dominant oscillatory component in the studied model signal $x(t)$, i. e. the oscillatory pattern corresponding to the frequency with max amplitude value. This situation is clearly presented in Fig. 2, (c) . As shown in Fig. 2, (d), the analysis of the first and second CWT skeletons dynamics allows to gain a better understanding of the real dynamics of the signal $x(t)$. However, we observe, firstly, the loss of information about "weak" oscillatory patterns with low amplitude. Secondly, the numerical description of each pattern dynamics shows a mix of various CWT skeletons, which complicates further analysis. Further, the results of the numerical analysis of model signal $x(t)$ based on the developed method of modified CWT skeletons estimation are presented in Fig. 2, (e). It can be seen that this technique correctly identifies in the time and frequency domains each of the oscillatory patterns being in the model signal $x(t)$.

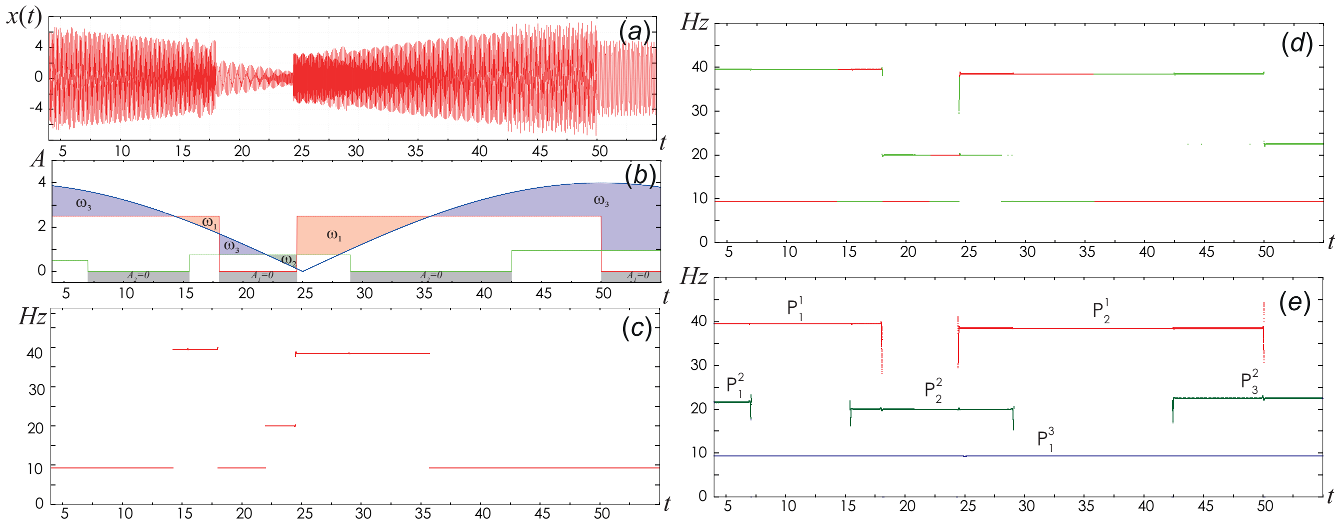

Figure 2. (a) – The model signal $x(t)$ (7); (b) – the time dependence of each amplitudes $A_1(t)$, $A_2(t)$ and $A_3(t)$ of oscillatory components; (c) – the time dependence of the first CWT - skeleton; (d) – the time dependences of the first and second CWT skeletons; (e) – the time dependence of modified CWT skeleton, estimated in the proposed method.

We demonstrate the results of the proposed method in processing on an ECoG fragment. Figures 3, (a) and (b) show the results using the classic and modified skeleton method. The first classic approach allows us to clearly identify the first three dominant frequency components of the analyzed signal in each time moment . However, taking into account only dominant frequencies at each moment does not allow us to trace the evolution of these frequency components over time.

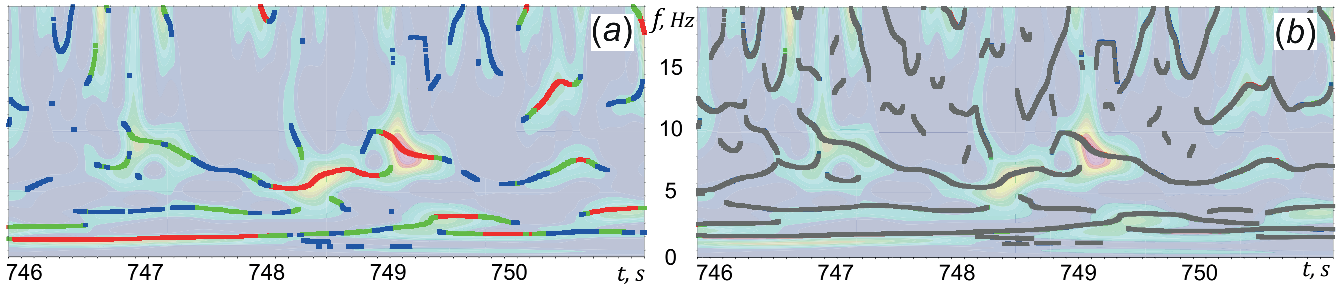

Figure 3. Schematic illustration shows using the classical CWT skeleton method (a) and the proposed the modified skeleton method (b) to detect frequency patterns. Evaluation results are given for an ECoG fragment lasting 5 s, which was recorded in rats during quiet wakefulness.

Figure 3 (b) allows us to see that the oscillatory activity dynamically develops over time and formes oscillatory patterns with various durations, changing in the frequency and amplitude zones, up to complete destruction. Obviously, the our exstension of the wavelet-technique used to process the ECoGs as a whole has outstanding sensitivity and allows identifying activity of a very low magnitude. In this situation, a rational analysis of the evolution of the entire frequency spectrum is adopted to gain a detailed understanding of signal oscillatory structure. We can observe patterns of oscillatory activity of fluctuating amplitude in different frequency bands. Moreover, the using of this method allows for a simple quantitative assessment of signal patterns for a specific frequency range and time interval.

Appendix C. Experimental Data Processing

We divided the entire significant frequency range of the ECoG signal into 6 intervals: Δf_1 [1; 2,5] Hz, Δf_2 [2,5; 4,5] Hz, Δf_3 [4,5; 6,5] Hz, Δf_4 [6,5; 9] Hz, Δf_5 [9; 12] Hz, Δf_6 [12; 14] Hz. After that, for each frequency interval Δf, we estimate the time-normalized number of $N_{\Delta f}$ patterns with an average frequency f_{md} falling in this frequency interval Δf, namely:

$$N_{\Delta f} = \frac{m}{t_2 - t_1} \text{ for } \forall P, \text{ if } f_{md} \in \Delta f, \tag{8}$$

where m is the total number of oscillatory patterns P per one identified awake or sleep episode, t_1 is the awake/sleep episode start time, and t_2 is the awake/sleep episode end time.

Also, for each episode of wakefulness/sleep, we calculate the average normalized time length of the "life", duration $T_{\Delta f}$ of the pattern according to the following equation:

$$T_{\Delta f} = \frac{\sum_{k=1}^{m} \tau_k}{t_2 - t_1} \text{ for } \forall P, \text{ if } f_{md} \in \Delta f, \tag{9}$$

where τ_k is the time duration of the kth oscillatory pattern with an average frequency f_{md} lying within the boundaries of a certain frequency range Δf during a certain awake/sleep period.

References

1. Runnova, A. *et al.* Automatic wavelet-based assessment of behavioral sleep using multichannel electrocorticography in rats. *Sleep Breath.* 1–8 (2021).
2. Pavlov, A. *et al.* Extended detrended fluctuation analysis of sound-induced changes in brain electrical activity. *Chaos, Solitons & Fractals* **139**, 109989 (2020).
3. Pavlov, A. *et al.* Extended detrended fluctuation analysis of electroencephalograms signals during sleep and the opening of the blood-brain barrier. *Chaos* **30**, 073138 (2020).
4. Semyachkina-Glushkovskaya, O. *et al.* Mechanisms of sound-induced opening of the blood-brain barrier. *Adv Exp Med Biol* **1269** (2020).
5. Semyachkina-Glushkovskaya, O. *et al.* Music/sound opens the blood-brain barrier: a readily available approach to brain drug delivery and therapy of brain diseases. *Proceeding Royal Soc. B.* (2020).
6. Koletar, M., Dorr, A., Brown, M., McLaurin, J. & Stefanovic, B. Refinement of a chronic cranial window implant in the rat for longitudinal in vivo two-photon fluorescence microscopy of neurovascular function. *Sci. Reports* **22**, 5499 (2019).
7. Torresani, B. Continuous wavelet transform. *Savoire, Paris* **675**, 676 (1995).
8. Hramov, A. E., Koronovskii, A. A., Makarov, V. A., Pavlov, A. N. & Sitnikova, E. *Wavelets in neuroscience* (Springer, 2015).
9. Pavlov, A. N. *et al.* Wavelet analysis in neurodynamics. *Physics-Uspekhi* **55**, 845 (2012).
10. Dai, Z. *et al.* EEG cortical connectivity analysis of working memory reveals topological reorganization in theta and alpha bands. *Front. human neuroscience* **11**, 237 (2017).
11. Braun, U. *et al.* Dynamic reconfiguration of frontal brain networks during executive cognition in humans. *Proc. Natl. Acad. Sci.* **112**, 11678–11683 (2015).
12. Makarov, V. V. *et al.* Betweenness centrality in multiplex brain network during mental task evaluation. *Phys. Rev. E* **98**, 062413 (2018).
13. Sitnikova, E., Hramov, A. E., Grubov, V. & Koronovsky, A. A. Age-dependent increase of absence seizures and intrinsic frequency dynamics of sleep spindles in rats. *Neurosci. journal* **2014** (2014).
14. Sitnikova, E., Hramov, A. E., Grubov, V. & Koronovsky, A. A. Time-frequency characteristics and dynamics of sleep spindles in WAG/Rij rats with absence epilepsy. *Brain research* **1543**, 290–299 (2014).
15. Runnova, A. E., Zhuravlev, M. O., Pysarchik, A. N., Khramova, M. V. & Grubov, V. V. The study of cognitive processes in the brain EEG during the perception of bistable images using wavelet skeleton. In *Dynamics and Fluctuations in Biomedical Photonics XIV*, vol. 10063, 1006319 (International Society for Optics and Photonics, 2017).
16. Maksimenko, V. A. *et al.* Visual perception affected by motivation and alertness controlled by a noninvasive brain-computer interface. *PloS one* **12**, e0188700 (2017).
